# A qualitative study on the lived experiences of individuals with end-stage kidney disease (ESKD) accessing haemodialysis in Northern Ghana

**DOI:** 10.1186/s12882-024-03622-x

**Published:** 2024-05-31

**Authors:** Edward Appiah Boateng, Aduni Amina Iddrisu, Joana Kyei-Dompim, Philemon Adoliwine Amooba

**Affiliations:** 1 Department of Nursing, Kwame Nkrumah University of Science and Technology, Kumasi, Ghana; 2 Community Health Nurse’s Training College, Navrongo, Ghana

**Keywords:** End-stage kidney disease, Haemodialysis, Chronic kidney disease, Renal replacement therapy, Northern Ghana

## Abstract

**Background:**

Haemodialysis is Ghana’s most common form of renal replacement therapy for end-stage kidney disease (ESKD). However, limited research has explored the experiences of individuals with ESKD receiving haemodialysis in relatively poorer regions, especially in the northern part of the country. This study explored individuals’ experiences with accessing haemodialysis in northern Ghana and was guided by Levesque’s conceptual framework of access to healthcare.

**Methods:**

The study utilized a phenomenological design, and participants were recruited through the purposive sampling method. Individuals with ESKD receiving haemodialysis for at least three months were interviewed using a semi-structured interview guide. Trustworthiness was ensured through credibility, transferability, dependability, and confirmability.

**Results:**

Most of the participants had a history of hypertension that progressed to ESKD due to poor management practices – mainly purchasing antihypertensive drugs over the counter without visits to the hypertensive clinic contributed greatly to the delay in diagnosing ESKD in northern Ghana. The geographical location of the dialysis centre and limited dialysis machines were the key barriers to adequate access to dialysis. Many participants had two dialysis sessions per week instead of thrice a week. Some were even having one session per week due to the distance from their residence to the dialysis centre and the costs involved.

**Conclusion:**

Access to haemodialysis for individuals with ESKD in the northern part of the country is relatively limited in many ways compared with that in the southern part of the country. Health policies should include funding for haemodialysis and a collaboration with pharmaceutical companies to consider manufacturing dialysis concentrates to reduce the cost. Additionally, there should be deliberate efforts to design and implement programs to promote ESKD awareness in the country, especially in relatively poorer regions.

**Supplementary Information:**

The online version contains supplementary material available at 10.1186/s12882-024-03622-x.

## Background

Approximately 800 million individuals live with chronic kidney disease (CKD) worldwide. This condition disproportionately affects low- and middle-income countries (LMICs), where both prevalence and death rates are considerably high due to inadequate resources to deal with its consequences [1]. CKD inevitably progresses to end-stage kidney disease (ESKD), and when this occurs, there is a need for renal replacement therapy (RRT**)** to save lives [2]. The most common available RRT modality in Ghana is haemodialysis. Meanwhile, haemodialysis facilities are centred mainly in teaching hospitals in the country, with other administrative regions entirely deprived of any form of RRT [3]. However, the number of patients with ESKD who require RRT in Ghana is increasing due to the increasing prevalence of hypertension [4].

The northern part of Ghana has only one publicly funded dialysis centre, which is situated in the only teaching hospital in the area, with fewer than ten functioning haemodialysis machines [3]. Although there is no reported prevalence of ESKD in northern Ghana, the estimated number of people living with hypertension as well as those with ESKD requiring dialysis suggest an unmet need. Those diagnosed with ESKD have to travel a long distance to receive dialysis service as well as pay out-of-pocket since haemodialysis is not covered by Ghana’s National Health Insurance Scheme (NHIS) [3, 5]. Understanding the experiences of individuals diagnosed with ESKD in accessing RRT in a setting with limited haemodialysis centres and considered disproportionately poor within the country is critical in addressing their primary concerns while advocating for appropriate policy changes. This study aimed to provide a deeper understanding of the day-to-day issues that individuals living with ESKD in northern Ghana face while accessing RRT to influence policy decisions within the country.

## Conceptual framework

**Fig. 1:**
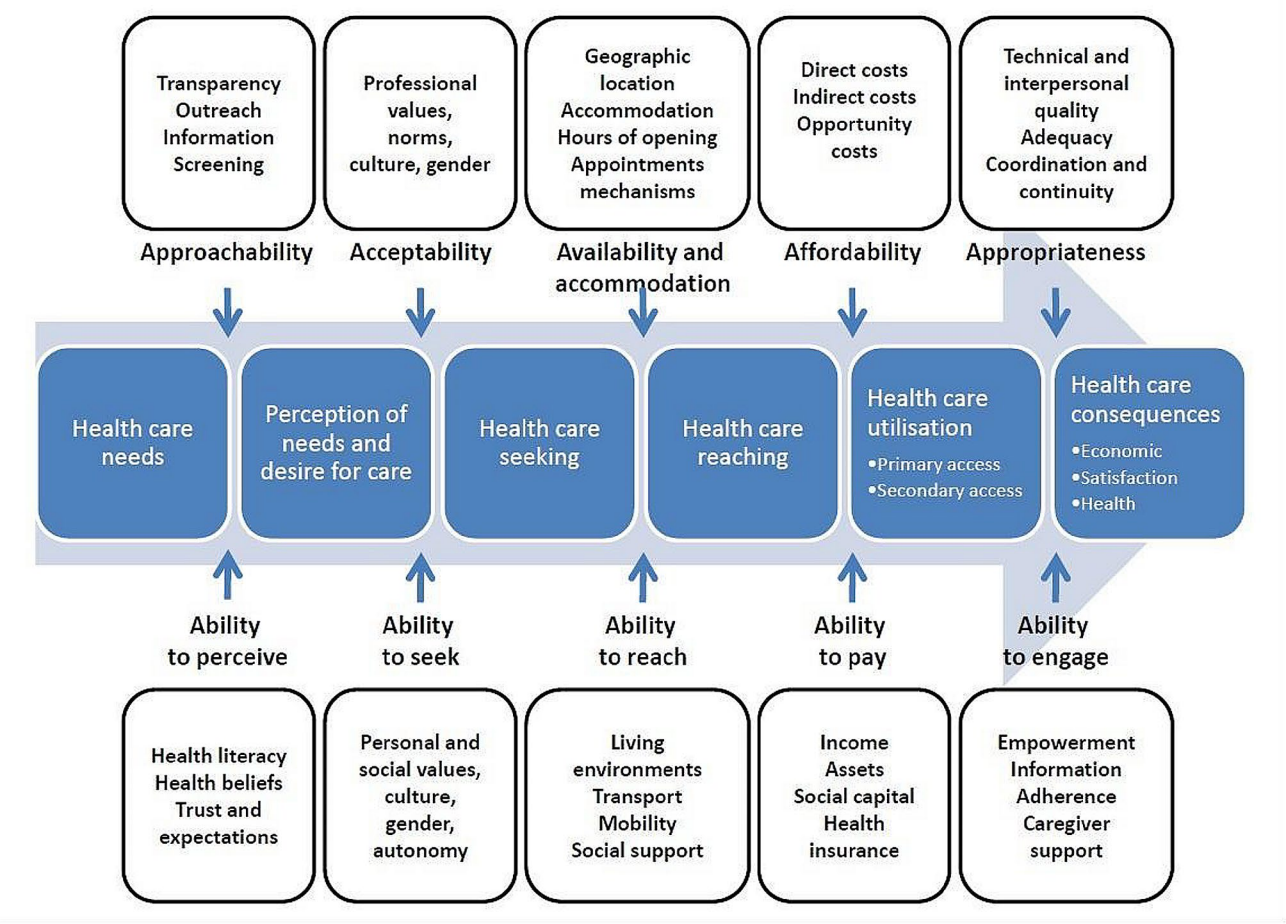
Levesque et al. (2013) Conceptual framework of access to healthcare

This study was guided by Levesque’s conceptual framework of access to healthcare [6]. It is a patient-centred approach that describes access to health services as a six-step process: health needs, perception of these needs, seeking health, reaching healthcare, service utilization, and health consequences (Fig. 1). The framework has five dimensions – approachability, acceptability, availability and accommodation, affordability and appropriateness – and their matching personal abilities (ability to perceive, ability to seek, ability to reach, ability to pay, and ability to engage).

Approachability refers to the situation where persons with health needs are aware that certain types of services exist and how to access them. The ability to perceive the need for care is the corresponding ability. Acceptability relates to the cultural and societal components that influence people’s willingness to accept certain aspects of services. The ability to seek healthcare is linked to personal autonomy and the capability to choose to seek care. Availability refers to the physical presence of health resources with adequate capability to produce services. The ability to reach health care relates to personal mobility and availability of transportation and occupational flexibility that would enable an individual to physically reach service providers. Affordability refers to a person’s ability to generate economic resources such as income to pay for healthcare services without depleting other assets. Its related ability is the capacity of a person or a household to pay for healthcare services. Appropriateness refers to how well a service satisfies a client’s needs. A client’s ability to participate in and commit to health care is governed by his or her capacity and willingness to participate in and commit to care.

## Methods

### Setting

This study was carried out at the only publicly funded dialysis centre in the only teaching hospital in northern Ghana that serves the Upper West and East regions, the Northern Region, the Savanna Region, some parts of the Bono East Region, and the northern part of the Volta Region. The dialysis centre, at the time of data collection, had eleven dialysis machines, with only six of them functional. The centre was managed by one nephrologist, assisted by three medical doctors, 2 nephrology nurses and 15 general nurses. The centre can carry out up to five sessions daily, dialyzing up to 30 individuals a day, depending on the number of individuals requiring dialysis, either planned or as emergencies. The regular working hours, however, are between 5 am and 1 am the next day, having staff working in three different shifts. The unit provides services each day of the week. Some individuals may report late or miss their scheduled sessions because of financial factors or some extenuating circumstances, making it difficult to strictly adhere to scheduled dialysis sessions at all times. The majority of these individuals reside in towns that are far from the centre, with travel times between three and seven hours to reach the centre.

### Study design and data collection

This study employed a qualitative research design using a phenomenological approach. Purposive sampling was used to recruit the study participants, with sample size informed by the depth of information provided by participants as well as the expected timeframe for the completion of the study [7]. The inclusion criteria for this study were individuals who were between 18 years and 75 years old, could communicate in English or Hausa, had been diagnosed with ESKD, and had received haemodialysis for at least three months. Those who were critically ill to participate were not interviewed. The selection of the participants was based on their experiences regarding accessing haemodialysis in Northern Ghana and their willingness to participate in the study.

The data collection took place from May to August 2022. After obtaining appropriate approvals, the nurse-in-charge of the dialysis unit introduced the researchers to potential participants. The study’s objectives were discussed with them, and all their questions and concerns were addressed. Individuals who agreed to participate signed or thumb-printed the consent form. All those who were approached agreed to participate in the study except for one who declined to participate without a reason.

Twelve individuals who met the inclusion criteria were recruited. Data were collected through semi-structured, face-to-face interviews with an interview guide developed in line with the study’s objectives and guided by the conceptual framework of access to healthcare [6]. Participants were interviewed and audio-recorded when they were receiving haemodialysis. It was ensured that no person other than AAI and the participant were present during each interview session, with no other person within the hearing reach of their interactions. The interview guide was assessed following the first two interviews to ensure that it was clear and appropriate for this study. No significant revisions were made to the guide after the first two interviews, so they were added to the main study. Probes were integral to the interview process, as they are essential in generating quality data for the study [8]. Field notes were also taken during and after each interview to keep records of nonverbal cues observed. All interviews were conducted by AAI in English based on participants’ preferences, lasting between 45 and 62 min. All participants were interviewed once, with no repeat interviews.

### Data analysis

Reflexive thematic analysis was employed for this study [8, 9]. This involved data familiarization, initial coding, generating initial themes, reviewing themes, refining, defining and naming themes and writing the report. All authors were involved in transcribing the interviews, a process that enhanced familiarization with the data. The researchers repeatedly read each interview transcript while listening to the audio version to ensure its accuracy and marking ideas for coding, mainly led by AAI. The proofread transcripts were uploaded onto the NVivo 12 software version application for organization and management. AAI then read through the transcripts and identified segments of the data that could be assessed in a meaningful way regarding the research objectives and initially coded them. These codes were carefully examined, and patterns among them were identified to generate themes and subthemes that were in line with the thematic areas of the conceptual framework for the study. The identified themes were reviewed, and one that did not fit the thematic patterns of the conceptual framework was constructed as a separate theme. Indeed, the writing of the report does not just happen at the end of the analysis; very much happens concurrently [9]. However, the final part of this report writing yielded a coherent arrangement of themes to establish a logical connection between them.

### Ethical consideration

Ethics approval was obtained from the Committee on Human Research, Publication and Ethics (CHRPE), KNUST (CHRPE/AP/221/22) before the start of the study. Prospective participants were provided with copies of the participant information leaflet to read, and detailed explanations of the contents were provided before the start of the data collection. In-depth responses to all concerns were provided. Participating in an interview about one’s experience with a severe chronic condition could be emotionally stressful. In that regard, it was stated explicitly to all eligible participants that they were under no obligation to participate and that their involvement was entirely voluntary. They could choose not to answer a question if they felt it was intrusive or personal. Eligible participants who agreed to participate signed or thumb-printed the consent form. Participants’ information gathered during the study was treated as strictly confidential, and pseudonyms were used to ensure anonymity.

## Results

### Characteristics of participants

This study comprised twelve participants, with nine being male and three being female. Nine participants reported having only hypertension before being diagnosed with ESKD. The youngest participant was 30 years old, while the oldest was 59 years old. Eight participants lost their jobs because of ESKD. The characteristics of the participants are summarized in Table 1, while the main findings in relation to the J-F Levesque, MF Harris and G Russell [6] conceptual framework are summarized in Table 2.

**Table 1:** Characteristics of participants with ESKD in Northern Ghana

Participant code	Gender	Age (years)	Period on Dialysis	Dialysis Sessions/week	Comorbidity	Previous Occupation	Current Occupation
1	Female	50–54	7 years	Twice	HTN	Teacher	Teacher
2	Male	35–39	4 months	Once	HTN	Customer Service	Unemployed
3	Female	30–34	3 years	Twice	HTN	Records Assistant	Unemployed
4	Male	45–49	9 years	Twice	HTN	Accountant	Accountant
5	Male	35–39	17 months	Twice	HTN/DM	Trader	Unemployed
6	Female	30–34	12 months	Once	HTN	Nurse	Sick leave
7	Male	35–39	12 months	Twice	HTN	Teacher	Teacher
8	Male	30–34	6 months	Once	Unknown	Taylor	Unemployed
9	Male	50–54	5 months	Once	HTN	Mason	Unemployed
10	Male	45–49	5 months	Twice	HTN	Trader	Unemployed
11	Male	30–34	4 months	Twice	HTN	Unemployed	Unemployed
12	Male	55–59	18 months	Twice	Stroke/HTN/ DM	Trader	Unemployed

(HTN – hypertension; DM – diabetes mellitus)

**Table 2:** Summary of findings in relation to the J-F Levesque, MF Harris and G Russell [6] conceptual framework

Dimension	Finding in study	Corresponding ability	Example quotes
Approachability	Lack of previous knowledge on ESKD and haemodialysis	Gaining information at the renal clinic	“…. I had hypertension for about five years, but I had never heard of ESKD. It was when I was diagnosed that I got to know there is a disease like that.” **P3**
Acceptability	Denial of diagnosis	Desire to choose treatment that works	“I didn’t want to come to the hospital for treatment because I felt that was not the problem. I did not believe my kidneys had a problem. The sickness started with my chest, so when they told me my kidneys had a problem, I did not believe that. I was expecting that they treat my chest not my kidneys. **P5**
	The use of alternative medicine		“Initially, when the sickness started and the doctor said I should do dialysis, I refused and went to use local medicine, but my condition was not improving, so I came back to the hospital to start the dialysis.” **P10**
	Cultural beliefs	Having support network	“As a man, you should not just run to the hospital with any little problem… Additionally, I believe the local medicine works faster so we were treating locally until one morning I started vomiting uncontrollably and I was sent to the hospital.**” P4**. “… I always went to a friend who has a drug store [pharmacy shop] to buy painkillers. One day, I went there, and he checked my BP and said that it was high. He gave me some drugs to take, and I went back for more whenever it finished.” **P10**
Availability	Geographical Location of dialysis centre	Availability of transportation and willingness to relocate	“…. the money that you are using for transportation or rent here could have helped so that you do more dialysis. See, the money I am using to pay the rent, I could have used that to do dialysis. I do twice a week, but if I add the rent money, I could do three times”. **P3**
	Inadequate dialysis machines	Adapting to prolonged waiting times	“One problem is that the machines are few, so you wait longer before it gets to your turn. The number of people who come for dialysis is high, so the pressure makes the machines break down frequently”. **P11**
	Interruption of scheduled activities	Having occupational flexibilities	“I come for dialysis on Tuesdays and Fridays so on these days I mostly don’t go to work” **P1**
Affordability	Economic burden	Making financial/treatment adjustments	“The cost of the treatment has affected us a lot because at times I have delays in paying my children’s school fees”. **P4**
	Lack of insurance cover/government subsidy		“One of the biggest problems, when you have this condition, is money. The insurance [NHIS] does not cover it, so I have used all my business money for dialysis.” **P5** “…Even the two times a week we cannot afford it. As of now, I am doing it once a week. When I started, I was doing 2–3 times a week, but now, I am doing once a week because I cannot afford.” **P6**
Appropriateness	Satisfaction with care provided	Appreciating care received	“They take care of us well. When you have a problem, they try to help, and we relate cordially with each other. Sometimes, when you miss your scheduled dialysis, they call to determine why.” **P6**
Ways to improve access	Policymakers/government support	Exploring alternative options	“If the government subsidized the cost, we would not be losing a lot of people because of ESKD. ….when you come to the north, someone will start, then 3–6 months afterwards, you will hear that their families said they cannot continue with it again and the next thing is that they have passed on”. **P12**
	Establishing more dialysis centers		“If the government can make them [dialysis centres] available at district hospitals, it will be very helpful. Honestly, if they can even have 2 or 3 machines for a start, because for all the regions in the northern part of the country, it is only in [this centre], and this is very bad.” **P2**

### Approachability

#### Lack of previous knowledge of ESKD and Haemodialysis

Participants had limited knowledge of ESKD. Almost all participants were aware that they had hypertension or diabetes mellitus but did not know much about ESKD as a complication. They got to know the existence of ESKD and haemodialysis after they were diagnosed. However, once participants received education about the disease at the renal clinic, they gained the corresponding ability to perceive the need to receive some form of treatment for their disease condition.

### Acceptability

#### Denial of diagnosis

The lack of knowledge about ESKD contributed to an initial denial of the diagnosis and delayed initiation of dialysis among participants, even when they had been told about the available option.

### The use of alternative medicine

The delayed initiation of dialysis among participants partly resulted from the use of herbal medicine, as many participants believed that herbal medicines work fast and could cure any disease. However, some participants were driven to use alternative medicine because of the costs associated with biomedicine, perceiving the former as a cheaper option. In the case of denying the diagnosis or using alternative medicine, the desire to choose a treatment option that improves their quality of life was seen as the corresponding ability for acceptability. When they felt that pursuing alternative medicine or denying the diagnosis was not improving their condition, they considered choosing biomedicine.

### Cultural beliefs

The use of alternative medicine was also influenced by cultural beliefs, as some participants, particularly the men, believed that visiting the hospital demonstrates weakness and did not want to be seen frequenting the hospital. Due to this belief, those who did not want to use herbal medicines preferred to purchase drugs from pharmacy shops when they felt unwell, rather than visiting a hospital for treatment. Having supportive network was seen as the corresponding ability for this – when the situation became overwhelming for the individuals, their families stepped in to support them, including taking them to the hospital for biomedical care.

### Availability

#### Geographical location of the dialysis centre

Dialysis services in the northern part of the country are provided solely at the centre of this study. Participants who lived within the city expressed satisfaction with the establishment of a dialysis facility and did not view distance as a barrier to receiving dialysis. However, those who travelled from distant places to the facility frequently encountered challenges. They often could not make it to the facility at scheduled times for their dialysis appointments. Those who travelled over long distances to the centre reduced the frequency or skipped some dialysis sessions because the cost of transportation depleted the funds they had set aside for the dialysis. Some had to relocate to the city so they could be close to the dialysis centre. However, they felt that they could have used the money for the rent to have more dialysis sessions.

In some instances, some participants finished their dialysis session late in the night and could not travel home, so they had to spend an extra day, leading to the loss of more days for work or other productive activities, yet creating extra expenditure for them in such situations. This led to the situation where the availability of transportation or willingness to relocate to a place near the dialysis centre became a corresponding ability for accessing healthcare.

### Inadequate dialysis machines

Participants reported that there were insufficient numbers of dialysis machines, and the few available machines frequently broke down. This resulted in long waiting times for dialysis services. It is noteworthy that participants managed to stay over an extended period to receive haemodialysis, giving them access to this lifesaving treatment in the face of all the inadequacies.

### Interruption of daily activities

For the majority of participants, getting dialysis meant forgoing other daily activities such as going to work in order not to miss their dialysis appointment, especially in the face of the limited dialysis machines. Thus, their willingness to alter their schedules served as the corresponding ability to give them access to treatment.

### Affordability

#### Economic burden

Treatment costs put a lot of participants under financial constraints. The continual expenditure over a long period caused their households’ financial resources to deplete. According to participants, they could not bear the cost alone. They depended on their family members and friends for support. Hence, individuals and families, sometimes, struggled to pay their children’s school fees and other expenses.

#### Lack of insurance cover/Government subsidy

The health insurance scheme in Ghana does not cover the cost of dialysis for individuals with ESKD. Some participants reported using all their salary for treatment, while others used all their trading capital. Due to financial constraints, some participants were compelled to reduce the frequency or forego their scheduled dialysis appointments, which ultimately affected their quality of life but granted them the ability to access dialysis care, even if occasionally and not in line with conventional treatment expectations.

### Appropriateness

#### Satisfaction with care provided

Receiving care from clinicians who professionally deliver care and cater to patients’ needs is associated with having positive experiences with healthcare. The majority of participants expressed high levels of satisfaction with the professional care they receive, especially from the nurses and this sustained their interest to be active participants in the care being provided despite the numerous challenges described above.

#### Ways to improve access

##### Policy makers/Government support

Many individuals diagnosed with ESKD do not commence dialysis after knowing the costs involved. Participants suggested that the government could subsidize the cost by covering half of the entire cost while they bear the other half.

### Establishing more dialysis centres

Participants also suggested the need to establish dialysis centres in the regional and district hospitals to reduce travelling expenses and the pressure on the haemodialysis machines that causes their frequent breakdowns. These suggestions provided some assurance to participants that, if they continued accessing haemodialysis care, the suggested expectation may become reality for them at a point and create universal access to haemodialysis for all individuals with ESKD in all parts of the country.

## Discussion

### Characteristics of patients with ESKD

The data presented in this study highlight the day-to-day experiences of individuals with ESKD accessing treatment in the northern part of Ghana and enable suggestions to be made to improve accessibility to ESKD. All but one of the participants had hypertension before developing ESKD. This underscores the central role of hypertension in increasing the global prevalence of CKD and, particularly, as the leading cause of ESKD in sub-Saharan Africa [4, 10–12]. It is noteworthy that some participants in this study had their hypertension detected at pharmacy shops where they also purchased antihypertensive medicines, without utilizing the routine care and diagnostic services provided by hypertensive clinics within the country. This resulted in a missed opportunity for the early diagnosis of CKD, as these individuals did not have their kidney function monitored over time to slow or halt the progression of CKD to ESKD. The key findings of the study will now be discussed under the key dimensions of the conceptual framework of access to healthcare [6].

### Acceptability

While lack of or inadequate knowledge prevented individuals with symptoms and/or a diagnosis of ESKD from accessing care, it also prevented them from accepting dialysis as a treatment option in the initial stages. Participants mostly denied the diagnosis and delayed the early initiation of treatment, resulting in the late commencement of dialysis, which also contributed to poor prognosis. Perceptions of accessing healthcare in a hospital setting as ‘unmanly’ contributed to delays in diagnosing ESKD among some participants in this study, as such individuals did not have their kidney function monitored over time. Indeed, cultural and religious beliefs as well as the desire for a cure inform the use of herbal medicines [15–18]. However, the same are also instrumental in accessing biomedical care, especially when other alternatives do not produce the desired outcomes. it is noteworthy that other participants attributed their use of herbal treatments to their inability to pay the cost of dialysis, a key barrier to accessing treatment for ESKD that has already been reported elsewhere [5, 17, 18].

### Approachability

Generally, almost all participants were aware that they had hypertension, diabetes mellitus or both but had no idea it could lead to the development of CKD or ESKD. They only became aware of this after they were diagnosed with ESKD, and haemodialysis when it was mentioned as the existing form of treatment. This lack of awareness of ESKD and haemodialysis increased the tendency of delayed treatment, as many participants were diagnosed late and had to start haemodialysis urgently to preserve their lives. As a consequence, many individuals with ESKD do not get adequate time to plan and prepare for it, resulting in abrupt discontinuation of haemodialysis after a few weeks or months after initiating it. Providing individuals with ESKD with appropriate information is critical to supporting them in addressing their information needs and decision-making about their care. Indeed, C Guha, P Lopez-Vargas, A Ju, T Gutman, NJ Scholes-Robertson, A Baumgart, G Wong, J Craig, T Usherwood and S Reid [13] and JT Hughes, N Freeman, B Beaton, A-M Puruntatemeri, M Hausin, G Tipiloura, P Wood, S Signal, SW Majoni and A Cass [14] report that individuals with CKD feel apprehensive about their prognosis and lack understanding of their condition because they are not provided with adequate information.

### Availability

A previous report on the number of functioning haemodialysis machines in the study setting was three, but this had increased to six functioning out of a total of eleven haemodialysis machines at the time of data collection for this study [3]. Although this seems to be an improvement, it remains woefully inadequate as the number of people diagnosed with ESKD who require haemodialysis grows to outweigh the number of machines available. The prolonged waiting times caused by the growing number of people with ESKD receiving dialysis services in the facility, along with insufficient functioning haemodialysis machines and frequent breakdowns, are by far the biggest obstacles participants face when trying to access haemodialysis at the facility. Some of the participants were frequently dissuaded from attending dialysis sessions due to waiting times, which usually forced them to turn to the commonly available and less expensive herbal medicines instead of dialysis. This challenge, unfortunately, is a common theme in resource-restricted settings [18].

Participants who resided in the city with the dialysis centre expressed satisfaction with the location of the facility, as some could even walk to the facility for haemodialysis services. However, the majority of our participants travelled to the dialysis centre from remote areas and different regions. This frequently made it difficult for them to arrive at the facility on time for their haemodialysis sessions, and some had to travel a day before their scheduled sessions to avoid delays. This was, undoubtedly, more concerning and stressful for such individuals while adding to the cost of their treatment. Geographical location as a barrier to accessing renal services has been widely reported in resource-constrained settings, and that of Ghana is well documented [3, 5, 18–20]. Indeed, it has been shown that individuals who live closer to a healthcare centre have better health outcomes than those who live further away [21]. There are calls for dialysis centres to be established in regions that lack them to improve access [18, 20].

### Affordability

The cost of a haemodialysis session at the time of data collection was GHC300 (USD 25), although there has been a twofold increase, excluding the cost of heparin, which is sold at varied prices in different pharmacy shops. Consequently, families have to contribute money for their kin to stay on dialysis. This continual expenditure ultimately depletes their financial resources, resulting in reduced or missed haemodialysis sessions, which consequently affects their quality of life. Indeed, haemodialysis imposes a substantial financial burden on families and health systems, and the impact is heaviest in resource-constrained settings [22]. Thus, the impact of the cost of treatment on accessing haemodialysis sessions cannot be overemphasized, especially in such a relatively poorer, resource-constrained setting.

### Appropriateness

Participants had a positive experience with health professionals, notably nurses whom they described as “good and friendly” in providing care to them. Positive experiences with healthcare professionals were linked to receiving support from staff who professionally delivered care and catered to their needs.

### Strengths and limitation of the study

One significant strength of this study is the representation of the contextual gap in the literature – it is the first to explore the experiences of individuals with ESKD accessing RRT in northern Ghana. This provides rich, contextual data on seemingly neglected regions in the country in terms of access to RRT. The use of the conceptual framework by Levesque et al. (2013) allowed various facets of access to RRT to be explored in this study and added to the richness of data generated in this study.

In terms of a key limitation of this study, participants had to be receiving haemodialysis to be included in the study. The perspectives of those who had discontinued dialysis would have offered useful insights into factors that led to their withdrawal. However, it was challenging to identify these individuals because they were not followed up, and some were reported to have passed away.

### Reflexivity

Researchers are central to data collection and analysis in qualitative research, making reflexivity an important aspect of the research process [8]. EAB is a registered nurse and a university lecturer with fourteen years of research experience in the management of ESKD. His interest is in enhancing the quality of life and decision-making experiences of individuals with ESKD. AAI is a registered nurse and a nurse educator. Her active interest in the management of ESKD was piqued during her postgraduate studies in 2021. JKD is a registered nurse, a university lecturer, and a PhD candidate. She has a special interest in using qualitative research methods to address comprehensive health problems in Ghana and beyond. PAA is a registered nurse and a university lecturer. He has a special interest in using qualitative research methods to address comprehensive health problems in Ghana and beyond. All these authors brought their unique perspectives to the design and analysis of data generated in this study.

## Conclusion

This study has shed light on the experiences of people with ESKD by revealing the actual issues that they face while accessing dialysis in northern Ghana. Geographical location and distance to the dialysis centre were the greatest obstacles to adequate access to dialysis in this study. While participants were mostly having two dialysis sessions per week, some were having one session per week. The latter group could have had at least two sessions per week but for the extra cost and inconveniences brought about by the distance between their residence and the dialysis centre.

Hypertension plays a key role in the development of CKD in the Ghanaian setting. While attending a hypertension clinic alone may not prevent the development of CKD as a complication, it will provide access to a range of tools and services that will delay its development or progression. Therefore, finding innovative ways to encourage individuals to get professional help in managing hypertension and dissuade them from purchasing antihypertensive drugs over the counter without appropriate consultation will contribute to the early diagnosis and management of CKD.

Improved ESKD awareness creation and screening, particularly in a setting with limited resources, will equip individuals with adequate information about ESKD and improve approachability. Finding innovative ways to reduce the high costs associated with RRT would contribute to improving affordability, making RRT financially accessible to people who need it while preventing the depletion of their financial resources. Strengthening financial support by incorporating RRT into the NHIS or reducing the cost of dialysis will improve access to RRT for many individuals who need this therapy [3, 5]. Programs such as partial funding of chronic dialysis, and the government and pharmaceutical industries collaborating to produce materials for dialysis locally rather than importing will help reduce the high dialysis cost for affected individuals and their families. Private and government partnerships could also lead to the establishment of more dialysis centres across the country to improve the availability of RRT services.

Additionally, there is an urgent need to decentralize RRT services to regional and district hospitals to improve availability to individuals in various settings. This could reduce the additional expenses of travelling and increase dialysis frequency while reducing the inconveniences of having to relocate to improve access to RRT.

### Electronic supplementary material

Below is the link to the electronic supplementary material.

Supplementary Material 1

Supplementary Material 2

## Data Availability

The datasets used and/or analysed during the current study are available from the corresponding author upon reasonable request.

## Abbreviations

ESKD: End-stage kidney disease
LMICs: Low- and middle-income countries
NHIS: National Health Insurance Scheme
RRT: Renal Replacement Therapy
