# Supplementary material for: A qualitative study on the lived experiences of individuals with end-stage kidney disease (ESKD) accessing haemodialysis in Northern Ghana
Source: BMC Nephrol. 2024 May 31;25:186. doi: 10.1186/s12882-024-03622-x (PMC11143648; doi:10.1186/s12882-024-03622-x)
Supplement: Supplementary file 1 — Supplementary Material 1 [file 12882_2024_3622_MOESM1_ESM.doc]

**Manuscript:** A Qualitative Study on the Lived Experiences of Individuals with End-Stage Kidney Disease Accessing Haemodialysis in Northern Ghana

Edward Appiah Boateng, Aduni Amina Iddrisu, Joana Kyei-Dompim, Philemon Adoliwine Amooba¹

**Consolidated criteria for reporting qualitative studies (COREQ): 32-item checklist**

Developed from:

Tong A, Sainsbury P, Craig J. Consolidated criteria for reporting qualitative research (COREQ): a 32-item checklist for interviews and focus groups. *International Journal for Quality in Health Care*. 2007. Volume 19, Number 6: pp. 349 – 357

| **No. Item** | **Guide questions/description**  **Author response/explanation** | **Reported on Page #** |
| --- | --- | --- |
| **Domain 1: Research team and reﬂexivity** |  | Page |
| *Personal Characteristics* |  |  |
| 1. Interviewer/facilitator | Which author/s conducted the interview or focus group?  AAI | Page 7 |
| 2. Credentials | What were the researcher’s credentials? E.g. PhD, MD  EAB – PhD, RN  AAI – MPhil, RN  JKD – Mphil, RN  PAA – PhD, RN | N/A |
| 3. Occupation | What was their occupation at the time of the study?  EAB – registered nurse, university lecturer  AAI – registered nurse, nurse educator  JKD – registered nurse, university lecturer  PAA – registered nurse, university lecturer | Page 8 |
| 4. Gender | Was the researcher male or female?  EAB – Male  AAI – Female  JKD – Female  PAA – Male | N/A |
| 5. Experience and training | What experience or training did the researcher have?  EAB, PAA and JKD are are experienced qualitative researchers with a number of publications.  AAI had completed various courses on research methods. | N/A |
| *Relationship with participants* |  |  |
| 6. Relationship established | Was a relationship established prior to study commencement?  After obtaining appropriate approvals, the nurse-in-charge of the dialysis unit introduced the researchers to potential participants. The study's objectives were discussed with them and all their questions and concerns were addressed.. | Page 6 |
| 7. Participant knowledge of the interviewer | What did the participants know about the researcher? e.g. personal goals, reasons for doing the research  All potential participants met with the researchers and the study's objectives were discussed with them. All their questions and concerns were addressed. | Page 6 |
| 8. Interviewer characteristics | What characteristics were reported about the interviewer/facilitator? e.g. Bias, assumptions, reasons and interests in the research topic  These have been explained in the manuscript. | Page 8 |

| **Domain 2: study design** |  |  |
| --- | --- | --- |
| *Theoretical framework* |  |  |
| 9. Methodological orientation and Theory | What methodological orientation was stated to underpin the study? e.g. grounded theory, discourse analysis, ethnography, phenomenology, content analysis  This study employed a qualitative research design using a phenomenological approach. | Page 6 |
| *Participant selection* |  |  |
| 10. Sampling | How were participants selected? e.g. purposive, convenience, consecutive, snowball  Purposive sampling was used to recruit the study participants. | Page 6 |
| 11. Method of approach | How were participants approached? e.g. face-to-face, telephone, mail, email  Data were collected through semi-structured, face-to-face interviews with an interview guide developed in line with the study's objectives and guided by the conceptual framework of access to healthcare by Levesque et al. (2013). | Page 7 |
| 12. Sample size | How many participants were in the study?  This study comprised twelve participants, with nine being male and three being female. | Page 7, 9 |
| 13. Non-participation | How many people refused to participate or dropped out? Reasons?  All those who were approached agreed to take part in the study except for one who declined to participate without a reason. | Page 6, 7 |
| *Setting* |  |  |
| 14. Setting of data collection | Where was the data collected? e.g. home, clinic, workplace  Participants were interviewed and audio-recorded when they were receiving haemodialysis. | Page 7  . |
| 15. Presence of non-participants | Was anyone else present besides the participants and researchers?  It was ensured that no person other than AAI and the participant were present during each interview session, with no other person within hearing reach of their interactions. | Page 7 |
| 16. Description of sample | What are the important characteristics of the sample? e.g. demographic data, date  A summary of this has been provided. | Page 10 |
| *Data collection* |  |  |
| 17. Interview guide | Were questions, prompts, guides provided by the authors? Was it pilot tested?  The interview guide was assessed following the first two interviews to ensure that it was clear and appropriate for this study.  Probes were integral to the interview process as it is essential in generating quality data for the study | Page 7 |
| 18. Repeat interviews | Were repeat interviews carried out? If yes, how many?  All participants were interviewed once, with no repeat interviews. | Page 7 |
| 19. Audio/visual recording | Did the research use audio or visual recording to collect the data?  Participants were interviewed and audio-recorded when they were receiving haemodialysis. | Page 7 |
| 20. Field notes | Were ﬁeld notes made during and/or after the interview or focus group?  Field notes were also taken during and after each interview to keep records of non-verbal cues observed. | Page 7 |
| 21. Duration | What was the duration of the interviews or focus group?  All interviews were conducted by AAI in English based on participants’ preference, lasting between 45 and 62 minutes. | Page 7 |
| 22. Data saturation | Was data saturation discussed?  Purposive sampling was used to recruit the study participants, with sample size informed by the depth of information provided by participants as well as the expected timeframe for the completion of the study | Page 6 |
| 23. Transcripts returned | Were transcripts returned to participants for comment and/or correction?  No | N/A |
| **Domain 3: analysis and ﬁndings** |  |  |
| *Data analysis* |  |  |
| 24. Number of data coders | How many data coders coded the data?  AAI then read through the transcripts and identified segments of the data that could be assessed in a meaningful way regarding the research objectives and initially coded them. | Page 7, 8 |
| 25. Description of the coding tree | Did authors provide a description of the coding tree?  No | N/A |
| 26. Derivation of themes | Were themes identiﬁed in advance or derived from the data?  These codes were carefully examined and patterns among them were identified to generate themes and sub-themes which were in line with the thematic areas of the conceptual framework for the study | Pages 8 |
| 27. Software | What software, if applicable, was used to manage the data?  The proofread transcripts were uploaded onto the NVivo 12 software version application for organization and management. | Page 7 |
| 28. Participant checking | Did participants provide feedback on the ﬁndings?  No | N/A |
| *Reporting* |  |  |
| 29. Quotations presented | Were participant quotations presented to illustrate the themes/ﬁndings? Was each quotation identiﬁed? e.g. participant number  Yes. Participant codes were used to identify each quotation. | Pages 10 to 17 |
| 30. Data and ﬁndings consistent | Was there consistency between the data presented and the ﬁndings?  Yes | Pages 10 to 17 |
| 31. Clarity of major themes | Were major themes clearly presented in the ﬁndings?  Yes | Pages 10 to 17 |
| 32. Clarity of minor themes | Is there a description of diverse cases or discussion of minor themes?  Yes, there is a discussion of major and minor themes | Pages 17 to 21 |
