# Supplementary material for: A qualitative study on the lived experiences of individuals with end-stage kidney disease (ESKD) accessing haemodialysis in Northern Ghana
Source: BMC Nephrol. 2024 May 31;25:186. doi: 10.1186/s12882-024-03622-x (PMC11143648; doi:10.1186/s12882-024-03622-x)
Supplement: Supplementary file 2 — Supplementary Material 2 [file 12882_2024_3622_MOESM2_ESM.docx]

**INTERVIEW GUIDE**

A Qualitative Study on the Lived Experiences of Individuals with End-Stage Kidney Disease (ESKD) Accessing Haemodialysis in Northern Ghana

| **Levesque’s five dimensions for access to care** | **Primary questions** |
| --- | --- |
| Approachability | Tell me about ESKD and how it is developed?  How much did you know about ESKD before your diagnosis? |
| Acceptability | What factors do you believe make it more difficult or more likely for patients with ESKD to accept haemodialysis treatment?  How is ESKD treatment delivered, and what factors help or hinder acceptance of ESKD treatment?  Tell me if you have ever reflected on stopping or continuing dialysis? Why? |
| Availability | What resources are required for ESKD treatment?  Are there any barriers/challenges to starting and continuing treatment? Discuss them  Is there anything specific to the geographical region?  (Health facilities, shortages of supplies/machines/waiting time/staff) |
| Affordability | What factors affect access to haemodialysis in terms of human and financial resources?  Please tell me how you raise money to pay for your treatment? (Self, family, employer, friends, church or mosque or community) |
| Appropriateness | How satisfied are you are with the quality of care you receive?  How would you assess your communication with health providers you have encountered? (Nurses, doctors and other professionals)  Do they spend time talking to you and giving advice  How does the behavior or attitude of some health care providers affect your treatment? |
| Suggestions to improve access | From your experience, what could be done to make life easier for individuals with ESKD?  What important things are needed to assist patients in rural regions begin or continue their treatment -and why?  Is there anything else that you'd like to share? |
